# The Role of Vitamin D in Reproductive Health—A Trojan Horse or the Golden Fleece?

**DOI:** 10.3390/nu7064139

**Published:** 2015-05-29

**Authors:** Filip A. Dabrowski, Barbara Grzechocinska, Miroslaw Wielgos

**Affiliations:** Department of Obstetrics and Gynecology, Faculty of Medicine, Medical University of Warsaw, Starynkiewicza Sq. 1/3, 02-015 Warsaw, Poland; E-Mails: fil.dabrowski@gmail.com (F.A.D.); miroslaw.wielgos@wum.edu.pl (M.W.)

**Keywords:** vitamin D, infertility, polycystic ovary syndrome, *in vitro* fertilization, male infertility, endometriosis infertility, myoma infertility, premature ovary failure, ART

## Abstract

In the last decade, vitamin D was in the spotlight in many fields of research. Despite numerous publications, its influence on reproductive health remains ambiguous. This paper presents an up-to-date review of current knowledge concerning the role of cholecalciferol in human reproduction. It covers various infertility issues, such as polycystic ovary syndrome, endometriosis, myoma-induced infertility, male infertility, premature ovary failure and *in vitro* fertilization techniques. Vitamin D deficiency, defined as serum concentration of 25-hydroxycalciferol of less than 50 nmol/L, is commonly noted more frequently than only in fertility clinic patients. It is a global trend that is observed in all age groups. The results of original publications dated up to 2015 have been summarized and discussed in a critical manner. Most experts agree that vitamin D supplementation is a necessity, particularly in women suffering from obesity, insulin resistance or small ovarian reserve, as well as in men with oligo- and asthenozoospermia if serum concentration should fall below 50 nmol/L (normal range up to 125 nmol/L). High concentration of vitamin D and its metabolites in decidua during the 1st trimester suggests its important role in the implantation process and a local immunological embryo-protection. On the other hand, evidence-based research did not prove a significant difference so far in ovulation stimulation or embryo development depending on vitamin D level. In one of the publications, it was also found that vitamin D binding protein (VDBP) has a molecular similarity to anti-sperm antibodies, and another one concluded that both low (<50 nmol/L) and high (>125 nmol/L) concentration of vitamin D are associated with decreased number and quality of spermatozoa in semen. Vitamin D is definitely not a Trojan Horse in reproductive health, since there were no adverse effects reported for vitamin D intake of up to 10,000 IU/day, but to proclaim it the Golden Fleece, more evidence is needed.

## 1. Introduction

The role of vitamin D in fertility treatment has been recently described in some comprehensive reports [1,2]. Numerous publications proclaim this particle a panacea for various mental and somatic chronic diseases, while other advise caution in prescribing it to some groups of patients. What is the role of cholecalciferol in human reproduction? Should its supplementation become a golden standard in fertility treatment, or do we still need more evidence on its effect?

Today we know that, in a healthy woman, vitamin D serum concentration is higher than in patients suffering from polycystic ovaries syndrome (PCOS). Vitamin D activity pathways in patients with PCOS remain unknown, but cholecalciferol supplementation improves their insulin resistance and the effects of infertility treatment. Vitamin D stimulates anti-Müllerian hormone (AMH) production as well, which is highly correlated with ovarian reserve preservation. Likewise, in patients with vitamin D deficiency, a higher occurrence of uterine myomas is observed—another established reason for infertility. Moreover, it was found that a high concentration of calcidiol is related to greater endometriosis incidence, which was explained by diminishing elimination of endometrial cells that pass to the peritoneal cavity via ovarian reflux. Like females, male low (<50 nmol/L) and high (>125 nmol/L) vitamin D serum concentrations decrease not only spermatozoa count but also their progressive movement as well as increasing morphology abnormalities. Therefore, a clinician must be careful when prescribing vitamin D preparations, especially in male patients.

Background: Since classical development of medicine in 1920, Vitamin D (cholecalciferol, VD) was strongly associated with rickets, after observation that cod liver consumption leads to a regression in rickets symptoms [3]. At the verge of the 21st century, rickets was sporadically observed in Europe. However, in developing countries, it is still a common problem in the pediatric population [4,5]. Currently, researchers are attempting to reveal the non-classical influence of cholecalciferol on health. Lower vitamin D levels have been found in many autoimmune diseases, such as rheumatoid arthritis, systemic lupus erythematosus, systemic sclerosis, type 1 diabetes mellitus, multiple sclerosis, inflammatory bowel diseases, autoimmune thyroid diseases [6,7]. Since 2013, the database of the US National Library of Medicine National Institutes of Health (PubMed) has gained 2050 new publications about vitamin D and contains 62,427 articles regarding this subject. Research on the role of vitamin D in reproduction process modulation seems especially interesting; their conclusions presumably have a significant practical meaning in infertility treatment, a serious medical problem affecting up to 53 million people worldwide [8].

For the time being, we know that vitamin D exists in two forms: vitamin D_2_ (ergocalciferol) and D_3_ (cholecalciferol). In animals, D_3_ is synthesized in the skin from its derivate (7-dehydrocholesterol) in the presence of ultraviolet B radiation (UVB), while D_2_ is made in fungi and yeast. Vitamin D uptake in the common diet is of minor significance, although cholecalciferol can be found in sea fish fat and liver oil, while ergocalciferol in green plants and mushrooms. In serum, vitamin D is transported by vitamin D-binding protein (VDBP) to the liver and metabolized to an active form of 25-hydroxycalciferol (25[OH] D) [9]. An optimal level of vitamin D concentration in blood serum should range between 50 and 125 nmol/L [10], even though some environmental studies imply that the target level should be set much higher—up to 250, or even at 300 nmol/L [11,12]. Its chemical structure resembles steroid hormones and it acts likewise via nuclear receptor (VDR, vitamin D receptor). In the kidneys, 25[OH] D is decomposed by 1-α-hydroxylase (by CYP27B1) to an active form of 1,25-dihydroxyvitamin D [13].

**Figure 1:**
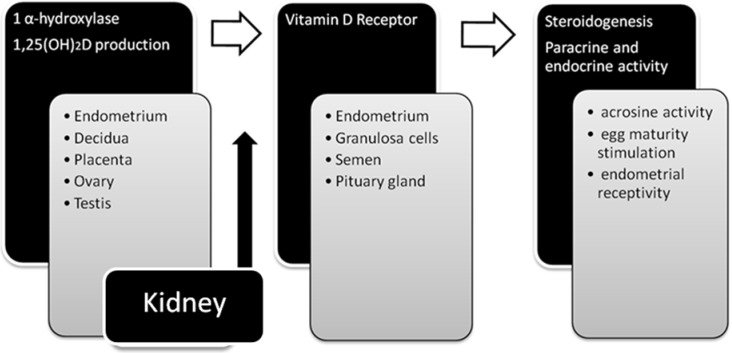
The role of vitamin D in the reproductive system (after: Grzechocinska B, Dabrowski FA, Cyganek A, Wielgos M. The role of vitamin D in impaired fertility treatment [2]).

The effect of vitamin D on ovarian granulosa cells responsible for steroidogenesis, as well as on immune system regulation, was established after finding 1-α-hydroxylase and VDR receptors in deciduae, placentas, ovaries, endometriums and pituitary glands, and was confirmed *in vitro* by demonstrating active metabolites of vitamin D such as 1,25[OH]_2_D and 24,25[OH]_2_D in mentioned tissues [14,15,16,17,18]. In male patients, the VDR was found in testicles and spermatozoa. It is known that 1,25[OH]_2_D is responsible for increasing intracellular Ca^+2^ concentration and the activity of acrosine, which is accountable for acrosome reactions that are essential for the fertilization process. An active form of 1-α-hydroxylase in sperm proves the existence of local synthesis of cholecalciferol and strongly suggests its paracrine effect on spermatogenesis as described in Figure 1 [19,20].

## 2. Methods

This article presents an up-to-date review of publications describing the function of cholecalciferol in reproduction and its place in infertility treatment. Under the key words “vitamin D, fertility”, 129 articles were found in the PubMed database, for “vitamin D, infertility”, another 106 were located (March 15th, 2015). Many of them contradict each other, or are at least incoherent. The results of original articles up to 2015 have been summarized and discussed in a critical manner to provide a consistent review.

## 3. Discussion

### 3.1. Polycystic Ovary Syndrome

PCOS is the most often diagnosed endocrinological disorder in women in their reproductive period, affecting 15%–20% of population, according to the European Society for Human Reproduction and Embryology standards [21]. Other commonly used guidelines are Rotterdam and Androgen Access Society criteria [22,23]. PCOS is very heterogonous and it is often impossible to determine the cause-effect relation between clinical symptoms and biochemical disorders [24].

Since our previous review [2], two new original papers have been published, explaining the significance of vitamin D in PCOS pathogenesis. They suggest that VD changes AMH production patterns in ovarian granulose cells and alters follicle stimulating hormone (FHS) sensitivity, possibly playing a role in ovarian follicle development. In healthy controls, 25[OH] D has shown to be positively correlated with AMH status, presenting seasonal variance [25]. In the second piece of research, VD supplementation was proven to increase serum levels of pro-inflammatory advanced glycation end products receptor (sRAGE), a particle binding those glycation end products (AGEs), which are known to be one of PCOS triggers (*p* = 0.03). At the same time, it decreases serum AMH levels (*p* < 0.001), which are often elevated in those women [26]. Obesity, VD accumulation in adipose tissue, and sunbathing avoidance due to hirsutism results in 65%–87% rate of VD deficiency in PCOS patients [27]. AMH level reduction and an increase of circulating sRAGE after VD_3_ exerted an anti-inflammatory action, which may lead to improved folliculogenesis in PCOS patients.

**Figure 2:**
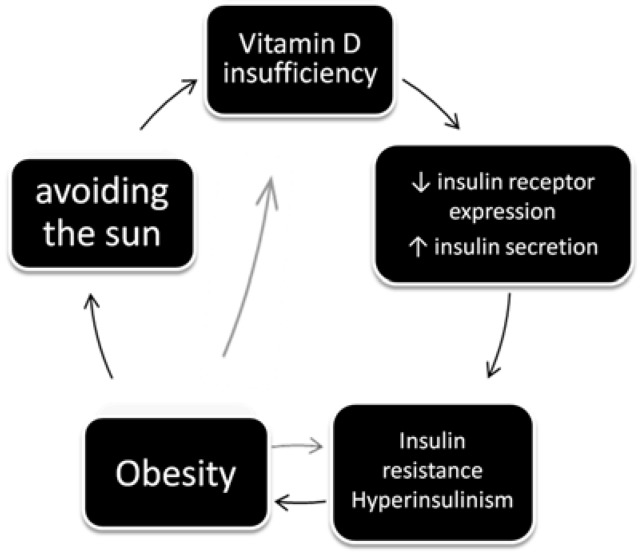
The effects and causes of vitamin D deficiency in women with PCOS (after: Grzechocinska B, Dabrowski FA, Cyganek A, Wielgos M. The role of vitamin D in impaired fertility treatment. [2].

VD serum levels are inversely proportional to blood pressure, lipid levels, insulin resistance and metabolic syndrome symptoms in PCOS patients [27,28,29,30]. Figure 2 shows how VD deficiency, together with obesity, may increase insulin resistance and deplete glucose transport through cellular walls—VD stimulates insulin receptor expression and insulin secretion [31].

In infertility studies, VD level was described as a predictor for ovulation stimulation success with the use of clomiphene citrate (CC). In 91 patients treated due to PCOS, ovulation was found in 57.1%, and pregnancy in 26.9% after using 50mg of CC. An unequivocal positive correlation was stated between VD_3_ level and the development of functional ovarian follicles, percentage of pregnancies, as well as proper body mass index (BMI) [28].

Another study demonstrated that, in obese women, the concentration of VD was considerably lower in patients with PCOS, which may prove its role in PCOS development [32]. In the light of provided evidence, VD supplementation was incorporated in the schemes of PCOS treatment.

### 3.2. Uterine Myomas

Last year gave us a new insight on VD-related gene polymorphism and the risk of uterine myomas (UM). It was earlier observed that lower concentration of VD is typical for women with fibroids (44.9 ± 19.2 *vs*. 51.9 ± 27.7 nmol/L, respectively, *p* = 0.010) and the risk (OR) of myomas in the VD deficiency group is 2.4 (95%; RR 1.2–4.9), (*p* = 0.16) [33]. It has long been known that African Americans are two to three times more prone to myomas than European Americans. This is concomitant with observational data showing that mean 25[OH] D concentrations in African Americans are near 40 nmol/L compared to about 65 nmol/L for white Americans [34]. A recent study on gene polymorphism in 2232 postmenopausal women has proven that two single nucleotide polymorphisms are significantly associated with UM. Gene rs12800438 is correlated with higher serum 25[OH] D levels, and gene rs6058017 - with lighter skin pigmentation [35]. Currently conducted animal studies provide data on probable therapeutic use of paricalcitol, a VD_3_ analog with lower calcemic activity, in uterine fibroid treatment [36]. So far, no human studies have been published concerning this compound, and ulipristal acetate remains our main weapon against UM.

### 3.3. Endometriosis

A paper published last year, entitled: “1-alpha, 25-dihydroxyvitamin D3 regresses endometriotic implants in rats by inhibiting neovascularization and altering regulation of matrix metalloproteinase” sets a new direction in VD research [37]. Up to this moment, researchers were focused on immunomodulating effect of vitamin D on endometriosis. It was confirmed that abnormally high VD concentration causes impaired elimination of endometrium cells passing to the peritoneal cavity via ovarian reflux [38]. In the study on 25(OH)D_3_, 1,25(OH)_2_D_3_ and Ca^2+^ levels, significantly higher concentrations of VD_3_ were confirmed in 87 patients with endometriosis (62.15 ± 36.9 nmol/L *vs*. 50.9 ± 29 nmol/L, *p* = 0.05). If serum concentration exceeded 70.3 nmol/L (75 percentile), the chance of endometriosis development was much higher, with OR = 4.8 (1.7–13.5) [39]. The concentration of vitamin D and Ca^+2^ were not dependent on menstrual cycle phase. Those two publications only seemingly contradict each other. An increased calcidiol level increases the risk of endometriosis occurrence, but in already existing cysts, it is a powerful inhibitor of neovascularization. No matter which angle we choose to look at for endometriosis, it is a disease caused by metabolic and biochemical imbalance. Therefore, determining VD status of those patients is so important. This complicated relationship is well described in some recent review publications [40].

### 3.4. Premature Ovarian Failure

Premature ovarian failure, defined as starting the menopausal period before the 40th year of life, can also be influenced by VD level. Except for the age factor, anti-Müllerian hormone (AMH) is well recognized as the biochemical marker of this syndrome [41]*.* After physiological fluctuations in childhood, AMH is stabilized at the age of eight and later begins to decrease from about the 25th year of life up to the beginning of menopause [42]. AMH is produced by ovary granulosa cells irrespective of stimulation with gonadotropins, but, as stated above, it may be triggered by VD supplementation. It is responsible for the stimulation of primary follicles in the ovaries and their susceptibility to FSH. In assisted reproduction, it is widely used as a parameter of ovarian reserve [43]. In a recent study, serum levels of vitamin D, steroid hormones, SHBG and ovarian reserve markers were determined in 73 non-obese, healthy, parous women. In linear regression analysis serum, vitamin D level positively correlated with total testosterone (*p* < 0.001) and free androgen index (*p* < 0.001). Authors suggest that VD may increase fertility through the modulation of androgen activity [44]. The direct effect of vitamin D on AMH level and follicle development was also confirmed in a recently published *in vitro* experiment [45].

The correlation between VD and AMH was confirmed in a multi-center study in patients over their 40th years of life. The authors of the study suggest a direct effect of vitamin D on AMH production, and thus longer maintenance of ovarian reserve in the patients with its higher concentration [46]. This data does not apply to PCOS patients with abnormally elevated AMH levels—as mentioned earlier, in this group, VD supplementation results with AMH level normalization [26].

### 3.5. Male Infertility Factor

Should males visiting infertility clinics be treated with VD preparations? The critical review of the available literature does not provide a consistent answer to this question. Recent animal studies proved that sperm count, motility, histological structure of testis, and spermatogenesis are more dependent on proper serum calcium and phosphorus levels rather than VD status [47]. In mice with VD 1-α-hydroxylase [1α(OH)ase(−/−)] deletion, serious fertility abnormalities were reversed with diet modification without VD supplementation. Authors state that the proliferation of spermatogenic cells was decreased with calcium-dependent down regulation of cyclin E and CDK2, and up regulation of p53 and p21 expression, which is not a direct effect of active vitamin D deficiency. Similar results have been reported in human subjects. According to a study on 300 men, Bloomberg concluded that 1,25(OH)D_3_ increased intracellular calcium concentration and sperm motility and induced the acrosome reaction in mature spermatozoa. VD serum levels were positively associated with sperm motility [48,49].

Earlier research suggested the adverse effect of vitamin D on fertility after a discovery of a molecular similarity of its transporting protein, the vitamin D binding protein (VDBP), to anti-sperm antibodies. Luckily, for the time being the *in vitro* experiments did not prove cross-reaction of specific immunoglobulin with VDBP [50].

Fertility cannot be the only concern of a physician consulting male patients. Infertile males with oligo-, astheno-, terato- and normospermia present a higher risk of osteoporosis and have lower bone mineral density (BMD) proportionally to testosterone and VD concentration (*p* < 0.01) in comparison with healthy controls at the same age. Significantly lower serum testosterone concentration is associated with lower BMD measured densitometrically in the lumbar spine and iliac bone (*p* < 0.05). The worst results were obtained in patients with bioavailable testosterone level below 11.6 nmol/l (*p* < 0.05). A strong, positive correlation between the concentration of VD and sperm motility and morphology was also found (*p* < 0.05). According to the recent studies [47], we can suggest that it was not a result of direct VD action, but its role in ion homeostasis [51]. The correlation between testosterone and VD has not been observed yet in another study conducted in Denmark on 307 fertile men. The lower serum VD level was also related to worse sperm parameters [52].

**Figure 3:**
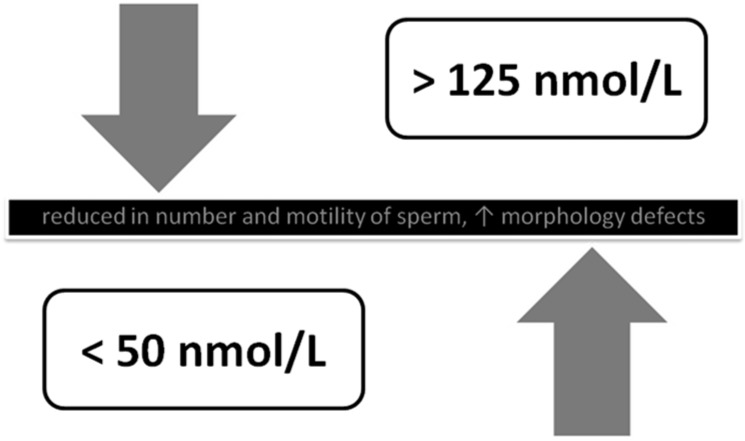
The influence of vitamin D on the activity and the morphology of sperm (after: Grzechocinska B, Dabrowski FA, Cyganek A, Wielgos M. The role of vitamin D in impaired fertility treatment [2]).

Another study describes what we have already learned from the paragraph on endometriosis. Ancient rules of *aurea mediocritas* are applicable once again. A study on hormonal factors in 147 males, selected from 170 volunteers, homogenous according to age (29 ± 8.5 years), BMI (24.3 ± 3.2) and stimulants intake demonstrated an abnormal (both low (<50 nmol/L) and high (>125 nmol/L) concentration of VD which negatively affected the sperm count, motility and morphology [53]. Dependence of male fertility on VD is depicted in Figure 3.

### 3.6. Vitamin D Effect on *In Vitro* Fertilization

VD insufficiency has been in the spotlight of *in vitro* fertilization (IVF) researchers for many years. Previously, most investigators wanted to determine the relationship between concentration of calcidiol in serum and follicular fluid. In 2010, 84 women were examined during IVF. Serum VD level correlated well with its follicular concentration (*p* = 0.001) and was inversely proportional to BMI of the patients (*p* = 0.04) [54]. As stated in the previous report [2], much higher concentrations of follicular VD were found in Caucasians (76.1 ± 32.3 nmol/L) compared to people of African descent (47.1 ± 21.2 nmol/L, *p* = 0.001). Today, we know it is directly associated with rs6058017 gene polymorphism [35]. The VD levels in patients with clinical pregnancy (30.95%) were significantly higher (*p* = 0.01) compared to those with early spontaneous abortion. Patients with high initial VD levels (267.8 ± 66.4 nmol/L) had a four time better chance (*p* = 0.02) for successful IVF procedure compared to the group with low VD levels (104.3 ± 21 nmol/L) [54].

In 2012, similar results were obtained from a group of 188 IVF-treated females in a tertiary academic center. This time, a significant difference in VD levels was found between Caucasian and Asian women (*p* = 0.001). It is interesting that in the Caucasian population, the chance of achieving clinical pregnancy (defined as fetal heartbeat visible in ultrasound at 7–8 weeks of gestation) increased with VD serum levels, while in Asians the reverse relationship was demonstrated [55]. After cross analysis including the number and quality of transferred embryos, it was shown that the patients with proper VD levels have a four time higher chance for a successful procedure.

Until 2014, it remained unknown which element—endometrial or egg quality—is more affected by VD deficiency. The answer was finally found in an experiment that involved 99 oocyte donor-recipient couples. In this study, VD levels were estimated in donor’s serum before the embryo transfer. As a result, adjusted clinical pregnancy rates were lower among VD-deficient recipients than among VD-replete recipients (37% *vs*. 78%). There were no differences among deeply deficient (VD < 50nmol/L) and VD insufficient (VD < 75nmol/L) recipients. Final live-birth rates were 31% among vitamin D-deficient recipients, compared with 59% among vitamin D-replete recipients. This very clever approach gave the authors of these, and of other similar studies from both Europe and the USA, reasons to state that their data strongly suggest that the effects of vitamin D may be mediated through the endometrium, not the follicle or oocyte [56,57].

### 3.7. Why is the Endometrium So Susceptible to VD Levels?

As described earlier [2], VD has a potent immuno-modulating activity towards lymphocytes T and the antigen-presenting cells. VD analog supplementation proved to be effective not only in fertility treatment but also in the prevention of type 1 diabetes in non-obese diabetic (NOD) mice and the prolongation of survival of syngeneic islets grafts [58].

High VD serum levels and their derivatives are found in human decidua from the 1st trimester of pregnancy, which strongly suggests its contribution in the implantation process and a local immunological preference of the embryo. The expression and activity of 1-α-hydroxylase (*CYP27B1*) was evaluated in *in vitro* experiments on decidua cell cultures collected in the 1st and 3rd trimesters [15]. It has been proven that the synthesis of VD is higher in the 1st trimester (41 ± 11.8 fMole/h/mg) compared to the 3rd trimester (8 ± 4.4 fMole/h/mg, *p* < 0.05). Quantitative analysis with RT-PCR showed higher gene expression of *CYP27B1* in both stromal cells CD10^+VE^ and CD10^−VE^ in the 1st trimester of pregnancy. The immunomodulating role of vitamin D was also confirmed by stimulation of NK cells (Natural Killer CD56^+ve^) isolated from the 1^st^ trimester decidua. After 28 hours of incubation with VD_2_ or VD_3_, the cells demonstrated lower production of granulocytes and macrophages colony stimulating factor, interleukin 6, Tumor Necrosis Factor, and an increase in mRNA expression for Cathelicidin Antimicrobial Peptide (CAP), which has a direct antibacterial activity [15]. As stated by the authors, the results of their study prove an increased production of active form of vitamin D in the first trimester decidua, which modulates reactions in a paracrine manner between the mother’s and the embryo’s immune system during the implantation. Disruptions of the process of major neovascularization can lead to serious pregnancy complications, including preeclampsia (PE) in further gestation. However, a clinical study that included 280 pregnant women did not demonstrate correlations between concentrations of VD and PE [59], probably due to its multi-causal etiology.

Some earlier experiments, in which the serum and follicular fluid VD levels were compared with the pregnancy rate, did not show the same results. In 22.6% of 221 infertile patients, the VD serum concentration was lesser than 25 nmol/L, in 70.1% it was within the range of 25–72.3 nmol/L, while in the remaining 7.2% it was higher than 75 nmol/L. The serum and follicular fluid VD levels were in significant correlation, with *p* < 0.0000, but fertilization (43.17%, 53.37%, 58.77%, respectively) and implantation percentages (17.33, 15.26, 18.75%, respectively) did not present any significant correlation (*p* > 0.5) [60].

Do we know how VD levels impact the oocyte quality? Examination for concentration of VD_2_ and glucose of follicular fluid in 101 patients treated with IVF-ICSI method reported poorer quality of embryos (*p* = 0.01) and a lower rate of clinical pregnancies (14.5%) in patients with VD levels exceeding 75 nmol/L. In the group with VD concentration between 50 and 75 nmol/L, success rate was 32.7% and in the VD-deficient group, 32.3% (*p* = 0.05). In the same study, patients with the highest concentrations of VD_2_ had the lowest glucose concentration in follicular fluid (*p* = 0.003) [61]. Meticulous observation should include seasonal and regional patterns. Seasonal variations in the number of pregnancies have already been noted both in spontaneous reproduction and in IVF. In a study of 188,075 cases, significant variations of semen characteristics have been observed with a clear improvement of sperm morphology in August [62]. The clinic’s geographical location (region) should also be analyzed, because the midyear UVB intensity and VD intake in diet are different between populations. UVB intensity is a positive predictor of live birth following fresh ET, whereas altitude and annualized average regional temperature have an inverse relationship with live birth ratio following fresh ET [63]. Complex evaluation of vitamin D activity mechanism on fertility requires further studies. Examining large groups of VD-deficient patients should not be a problem—most societies are VD-deficient either way.

Epidemiological studies have shown that VD deficiency increases not only infertility rate but also the risk of serious pregnancy complications, such as preeclampsia and preterm birth [64]. This is why even during the most physiological pregnancy vitamin D supplementation in the amount of 800–1000 IU daily is highly recommended [65]. Even though some negative outcomes with U-shaped associations were observed, with risks at both low and high levels [66], the recent meta-analysis did not find any association with high VD levels and negative health outcomes [67]. The supplementation should be continued during the breast feeding period even in larger doses of 6000IU daily in order to avoid deficiency in neonates [68]. During the preparation for pregnancy in VD-deficient females (with serum VD 50–125nmol/L), it is advised to supplement vitamin D prior to the first visit in a fertility clinic. This simple, inexpensive and safe treatment may prevent the need to use more invasive therapy. To consistently raise the blood level of 25(OH)D above 75 nmol/L at least 1500–2000 IU/day VD is required [69]. The doses for adults and elderly people of proper body weight, and pregnant and lactating women should not exceed 4000 IU daily, and the supplementation dosage recommended for pregnant women in winter and less sunny countries is 1500–4000 IU per day (37.5–50.0 μg/day) [10,66,70].

## 4. Conclusions

Current research on the role of vitamin D in fertility impairments, such as polycystic ovary syndrome, uterine fibroids, improper semen parameters and in the case of *in vitro* treatments and pregnancy failure, suggests it plays an important role in human reproduction processes. Vitamin D supplementation is advised in infertility therapy in both partners. Couples with serum concentration exceeding 50 nmol/L have a higher chance of conception, but this does not regard all patients, where especially males should be supplemented with vitamin D from the beginning of the therapy. Administration of vitamin D is recommended in the case of considerable deficiency, particularly in obese, insulin resistant women with low AMH levels, as well as in men with oligo- and asthenozoospermia.

Vitamin D is definitely not a Trojan Horse in reproductive health since there were no adverse effects reported for vitamin D intake of up to 10,000 IU/d [66], but to proclaim it the Golden Fleece, more evidence is still required.
